# Callous–Unemotional Traits and Conduct Problems in Children: The Role of Strength and Positive Characteristics

**DOI:** 10.3390/bs14070609

**Published:** 2024-07-18

**Authors:** Patrícia Figueiredo, Andreia Azeredo, Ricardo Barroso, Fernando Barbosa

**Affiliations:** 1 Laboratory of Neuropsychophysiology, Faculty of Psychology and Educational Sciences, University of Porto, Rua Alfredo Allen, 4200-135 Porto, Portugal; up20180889@fpce.up.pt (A.A.);; 2 Department of Education and Psychology, University of Trás-os-Montes and Alto Douro, 5000-801 Vila Real, Portugal; rbarroso@utad.pt; 3 Center of Psychology, University of Porto, Rua Alfredo Allen, 4200-135 Porto, Portugal; 4 U.North Psychology Consortium, Portugal

**Keywords:** callous–unemotional traits, conduct problems, positive characteristics, self-regulation, children

## Abstract

In recent decades, many researchers have focused on the development of Conduct Problems from childhood to adolescence. Understanding behavior problems also requires an understanding of well-regulated characteristics. Focusing our assessment on strengths makes it possible, on the one hand, to help children or adolescents with deficits in important areas (e.g., socio-emotional deficits) to develop emotional regulation skills and adapt their responses to different contexts. This study aims to understand the role of self-competence, self-regulation, empathy, and responsibility (strength variables) in the relationship between Callous Unemotional characteristics and Conduct Problems, with a sample of 236 children aged between 3 and 10 years (M = 7.51, SD = 1.63), through mediation analysis. In general, our findings suggest that self-regulation significantly explains the relationship between the callous dimension of the Inventory of Callous–Unemotional Traits and Conduct Problems, pointing out that this strength variable seems to act as a protective factor against the development of behavior problems. No other mediation effects were found, and these results are considered in light of some limitations.

## 1. Introduction

Callous–unemotional (CU) traits include characteristics such as arrogance, a lack of remorse and empathy, and being manipulative and deceitful [1,2], usually predicting a severe, aggressive, and stable pattern of behavior problems, specifically Conduct Problems [3]. There is evidence that these traits are uniquely associated with aggressive and severe behavior problems [4], with stability from late childhood to early adolescence [5,6], representing the affective component of adult psychopathy. Children who express these traits often do not feel empathy for others, lack remorse for the disruptive behaviors in which they engage, can be very manipulative, and express low levels of fearful inhibition [7].

Although Frick and White [8] suggest that CU traits show stability, these traits are not immutable [9,10]. In fact, CU traits do not follow the same developmental trajectory in all children. Previous studies provide evidence for individual variability in the development of CU traits (e.g., [11,12,13]), but few studies have assessed the factors associated with stability or changes in CU and their translation into Conduct Problems (e.g., [13,14]). This heterogeneity within CU traits might be associated with different developmental processes that can help either explain the co-occurrence or not of Conduct Problems. The association between CU traits and Conduct Problems can accumulate with important individual and contextual risk and protective factors, suggesting that in parallel with CU traits, there are factors capable of influencing the development of adaptive and maladaptive behaviors (e.g., [15,16]). In this way, further understanding of both the risk and protective factors that might contribute to the relation between CU traits and Conduct Problems in youth is needed.

Furthermore, although research in child and youth psychology has evolved over the last few decades, creating a greater emphasis on assessing individual strengths and positive characteristics, the focus on risk factors or diagnosing pathology is still very marked [17,18,19,20]. Due to the social changes that occur in childhood, as well as the experience of transition from the family environment to the school environment [21], this is a key period during which children develop socio-emotional skills that may affect their ability to learn how to interact with others and their well-being [22,23,24]. Alongside the risk factors for pathology, assessing individual positive characteristics can provide a broader understanding of individuals’ abilities that can be used effectively to reduce the likelihood of developing Conduct Problems. In recent decades, many researchers have focused on the development of aggressive, antisocial, and violent behavior [25]. Understanding Conduct Problems also requires understanding those who exhibit well-regulated emotions [26], such as self-control and proper abilities, to deal with negative emotionality are fundamental to preventing pathological behavior [27]. Indeed, a common feature of Conduct Problems is the inability to effectively regulate emotions and self-evaluations in different contexts [27,28].

Self-regulation is described as a phenomenon that begins at birth and develops into adulthood, gaining significance at all stages of an individual’s life [29,30]. This construct refers to the inhibition of behavior or self-control, whereas its absence indicates impulsivity [31]. Self-regulation, a complex framework linked to both positive and negative adjustment, encompasses components such as working memory, attention, and inhibitory control [30,32]. Inhibitory control, which involves the regulation of behavior, refers to the ability to suppress impulses to achieve goals [33,34]. This structure is important for emotional, social, and cognitive development [35]. Particularly during early childhood, self-regulation assumes an important role in controlling the magnitude of emotions [36,37], including emotions that trigger aggression or provoke sadness and anxiety [38]. Although emotions are inherently regulated, their regulation varies depending on the person and the context [39]. Children with poor emotion regulation skills are at greater risk of poor behavioral outcomes. These children have difficulty getting along with adults and peers and are at higher risk of future aggressive behavior problems, Conduct Problems, substance abuse, and psychopathy [40,41].

On the contrary, children with high emotional regulation tend to be more socially competent [41]. Even when young people show low empathy or psychopathic traits, high self-control can enable them to avoid Conduct Problems and other manifestations of antisocial behavior [42]. Most studies based on questionnaires of adolescents reveal that non-antisocial young people with high CU traits have greater self-regulation than young people who score high on both CU traits and Conduct Problems [43]. Thus, the ability to regulate behavior considering individual goals, potential actions [44], and strategic planning [45] can differentiate young people with positive personality traits with and without Conduct Problems. Moreover, social competence has been a popular topic for research because it is not only a key aspect of children’s development but also a significant indicator of their functioning in other domains, such as academic achievement [46] or externalizing and internalizing psychopathology [47]. As such, identifying mechanisms for variations in children’s social competence may inform early prevention efforts to promote their healthy development. Children who show higher levels of CU may have more difficulty in relational contexts because the deficits that these children experience are key to successful social interactions [48,49]. Although CU traits and social competence may emerge simultaneously from early ages [50,51], they are distinct albeit related constructs. While CU traits are a stable, dispositional factor [6,52], social competence may be more malleable and determined by many contextual and individual factors, including CU traits [50].

Additionally, CU traits are primarily characterized by affective deficits. In contrast, social competence encompasses a more diverse set of interpersonal skills, some of which may be influenced by individual differences in CU traits. Furthermore, studies have shown that knowing and managing emotions is important for positive social interactions and stable relationships [53]. Thus, emotional competencies are important for acquiring social skills, especially for developing prosocial behavior and empathy [54], reducing their involvement in aggressive interactions [55,56].

In recent decades, many researchers have focused on the development of Conduct Problems from childhood to adolescence. Understanding Conduct Problems also requires understanding well-regulated characteristics [26]. The ability to self-control and deal with negative emotions are fundamental characteristics for understanding both normative and pathological behaviors [57]. Focusing on the assessment of strengths makes it possible, on the one hand, to help children or adolescents with deficits in important areas (e.g., socio-emotional deficits) to develop emotional regulation skills and adapt their responses to different contexts [58]. Therefore, the main objective of this study is to understand the role of strength and positive characteristics in the relationship between CU traits and Conduct Problems. It was hypothesized that (a) the callousness and uncaring dimensions of CU traits are positively related to Conduct Problems and that (b) strength and positive characteristics, namely social competence, self-regulation, empathy, and responsibility, mediate the above relation, i.e., they can diminish the effect of CU traits, reducing the likelihood of Conduct Problems occurring.

## 2. Materials and Methods

### 2.1. Participants

The selection of participants for this study was conducted in two phases. First, children between 3 and 10 years of age were randomly selected in schools in the Northern region of Portugal, and informed consent was provided to the parents of the selected children. Parents who agreed to participate and completed an informed consent form were sent a questionnaire about the behavior and routines of their children. Once the consent forms and questionnaires were received from the parents, the child’s teacher completed similar questionnaires. This procedure resulted in a sample of 236 children (52% girls; 4% receiving special education support) with characteristics closely approximating the participating school districts (see Table 1 for additional information on sample demographics). All procedures were appraised and followed the ethical standards of the institutional ethics committee.

### 2.2. Instruments and Measures

#### 2.2.1. Inventory of Callous–Unemotional Traits—Teacher (ICU-Teacher; [59,60]; Portuguese Version Developed by Figueiredo et al. [61,62])

The ICU teacher is a teacher-report inventory composed of 12 out of 24 original items for assessing callous–unemotional traits. It is responded to on a 4-point Likert scale, ranging from 0 (=Not at all true) to 3 (=Definitely true), leading to a minimum score of zero and a maximum of 36. Higher scores on the total scale and subscales of this instrument indicate higher levels of CU traits. The first validation study of the ICU, by Essau et al. [59], found satisfactory to adequate internal consistency values, with Cronbach’s α of 0.64 and 0.73 for the unemotional and uncaring subscales, respectively, and 0.77 for the total score. In the present study, Cronbach’s alphas ranged between 0.83 and 0.85. The Portuguese versions were developed by Figueiredo et al. [61,62] and are organized into two factors—callous and uncaring—comprising a total of 11 and 12 items, respectively, for each version.

#### 2.2.2. Strengths and Difficulties Questionnaire (SDQ; Goodman et al. [63])

The SDQ is a 25-item screening instrument that can be administered to parents and teachers of children aged 4 to 17 or as a self-report in participants over 11 years old. The questionnaire covers common social, emotional, and behavioral functioning areas across five subscales: Emotional Symptoms, Conduct Problems, Hyperactivity/Inattention, Peer problems, and Prosocial Behavior. The students’ and teachers’ versions are almost identical, except that the wording is slightly different (the students assess themselves, while the teachers assess their students). For each statement, the participants (students and/or teachers) respond using a 3-point Likert scale, ranging from 0 (=Not True) to 2 (=Certainly True), so that “0” indicates no problems, and “2” indicates problems. The maximum score for each subscale is 10, and the maximum total score is 50. Although the SDQ was adapted for Portuguese by Fleitlich et al. [64], the study of psychometric proprieties was carried on by Simões (see [65]) and revealed that a five-subscale structure is adequate for the Portuguese version of the SDQ. In the present study, SDQ was filled out by parents, and only the Conduct Problems subscale was used, which revealed a Cronbach’s alpha of 0.70.

#### 2.2.3. Social–Emotional Assets and Resilience Scale—Teacher Form (SEARS-T; [58])

The SEARS-T consists of a scale measuring children’s and adolescents’ social-emotional competencies and assets. Social and emotional assets and resilience can be broadly defined as a set of adaptive characteristics important for success at school, with peers, and in the outside world. The SEARS-T comprises 41 items on a 4-point Likert scale ranging from 0 (=Never) to 3 (=Always). It is organized into four empirically derived scales, including the following: (a) self-regulation; (b) empathy; (c) social competence; and (d) responsibility. The self-regulation subscale assesses the child’s self-awareness, metacognition, intrapersonal perception, and self-management from the teacher’s perspective (e.g., *disagrees without fighting or arguing*). The empathy subscale reflects the teacher’s assessment of the child’s empathy with the situations and feelings of others (e.g., *feeling sorry when bad things happen to others*). The social competence subscale reflects how the teacher perceives the child’s ability to maintain friendships with peers, engage in effective verbal communication, and feel comfortable in peer groups (e.g., *feeling comfortable talking to different people*). The responsibility subscale refers to the teacher’s assessment of the child’s ability to take responsibility, behave consciously, and think before acting (e.g., *making good decisions*). The Portuguese version by Figueiredo et al. [66], which comprises 40 items, revealed very high internal consistency for the four scales—responsibility, empathy, self-regulation, and self-competence—with Cronbach’s α of 0.94, 0.92, 0.95 and 0.92, respectively. In the present study, Cronbach’s α range was between 0.73 and 0.95.

### 2.3. Statistical Analyses

All analyses were performed using SPSS Version 28.0 for Windows (IBM, 2020), using a significance level of 0.05. Initially, the assumption of normality was assessed using the Shapiro–Wilk test, and when violated, this was complemented by analysis of the asymmetry (Sk) and kurtosis (Ku) coefficients. Since the absolute values of these coefficients varied between 2 and 7 [67], parametric tests were always used. The sphericity assumption was assessed using the Mauchly test, and when this assumption was violated, the Greenhouse–Geisser correction was applied, and the epsilon value (Ɛ) was reported. Preliminary statistical analyses were performed for all variables, including descriptive statistics and Pearson correlations. The Conduct Problems subscale of SDQ was entered as the dependent variable into a mediation analysis, resorting to the PROCESS macro for SPSS [68], which is a computational tool run within SPSS to generate mediation, moderation, and conditional process analyses [68]. CU trait variables (namely callous and uncaring scores of the ICU scale) were considered independent, and strength variables (specifically self-regulation, empathy, social competence, and responsibility scores of the SEARS scale) were considered mediators. All indirect effects were examined using bootstrapping analytical methods to estimate a bias-corrected 95% confidence interval (CI) by taking 5000 bootstrapped samples [69]. Confidence intervals of the conditional indirect effect that did not contain a value of zero indicated significant mediation effects. Complete mediation was considered to occur when path c’ (the direct effect) was equal to 0, suggesting that the association between X and Y was fully accounted for by the mediators, while partial mediation was considered when path c’ was a value other than 0 but significantly less than path c [69].

## 3. Results

Descriptive statistics for the variables of interest are provided in Table 1. Correlations between CU traits and Conduct Problems were examined, revealing that both callous, r = 0.21, *p* = 0.003, and uncaring scores, r = 0.34, *p* < 0.001, were positively related to Conduct Problems, as was the total score of ICU, r = 0.32, *p* < 0.001. In addition, both CU traits and Conduct Problems were negatively associated with strength and positive characteristics, namely social competence, empathy, self-regulation, and responsibility (see Table 2). These results support the further examination of strength variables as mediators in the relationship between CU traits and Conduct Problems.

### Mediation Effects of Strength and Positive Characteristics

**Figure 1:**
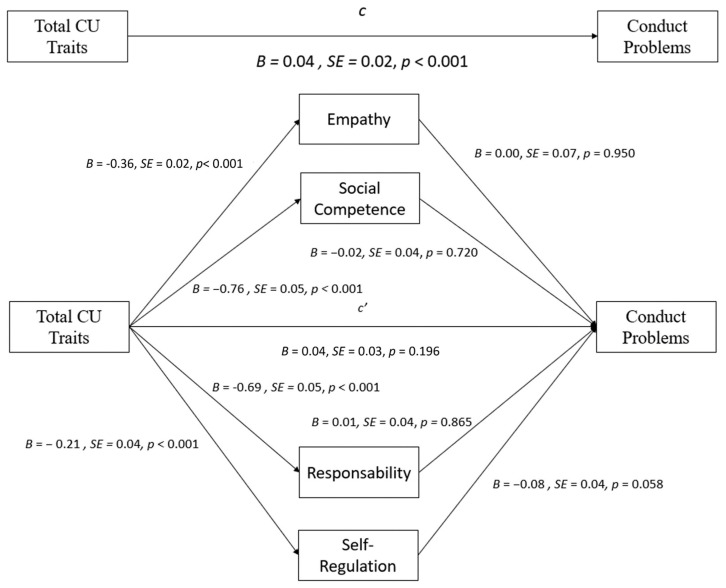
Mediation effects of strength variables on the relationship between total CU traits and Conduct Problems.

Three mediation models were computed to examine the strength and positive characteristics as possible mediators of the relationship between CU traits, namely total CU traits, callous, uncaring, and Conduct Problems. In the model, considering the total score of the ICU traits as the independent variable (Figure 1), bootstrapping analyses revealed the total effect of CU traits on Conduct Problems, b = 0.09, *p* < 0.001. Empathy, responsibility, self-regulation, and social competence did not significantly mediate the relation between total CU traits and Conduct Problems (all *p* > 0.05). It was verified that the total ICU score predicted all strength variables (all *p* < 0.001), but none of the said variables significantly predicted Conduct Problems (all *p* > 0.05). The results from a multiple mediation analysis testing empathy, responsibility, self-regulation, and social competence as mediators in the relation between total CU traits and Conduct Problems are presented in Table 3 and Figure 1.

**Figure 2:**
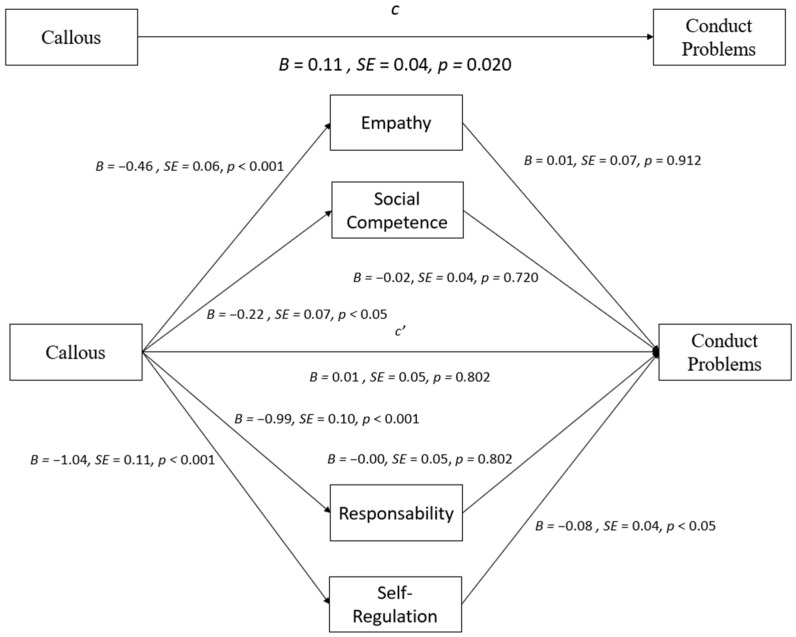
Mediation effects of strength variables on the relationship between the callousness subscale and Conduct Problems.

Taking the callous subscale score as the independent variable (Figure 2), bootstrapping analyses revealed the total effect on Conduct Problems, b = 0.11, *p* = 0.020. Self-regulation significantly mediated the relation between the callous subscale and Conduct Problems, such that higher scores in the callous subscale were associated with higher scores in the Conduct Problems subscale through the effects of decreased Self-regulation at BaC 95% CI [−0.162, −0.007]. Empathy, responsibility, and social competence subscales were not significant mediators in the relation between callous scores and Conduct Problems (all *p* > 0.05). The scores of the callous subscale predicted all strength and positive characteristics (all *p* < 0.05), but none of these variables significantly predicted Conduct Problems (all *p* > 0.05), except Self-regulation (*p* < 0.05). The results from a multiple mediation analysis testing empathy, responsibility, self-regulation, and social competence as mediators in the relationship between callous and Conduct Problems are presented in Table 3 and Figure 2.

**Figure 3:**
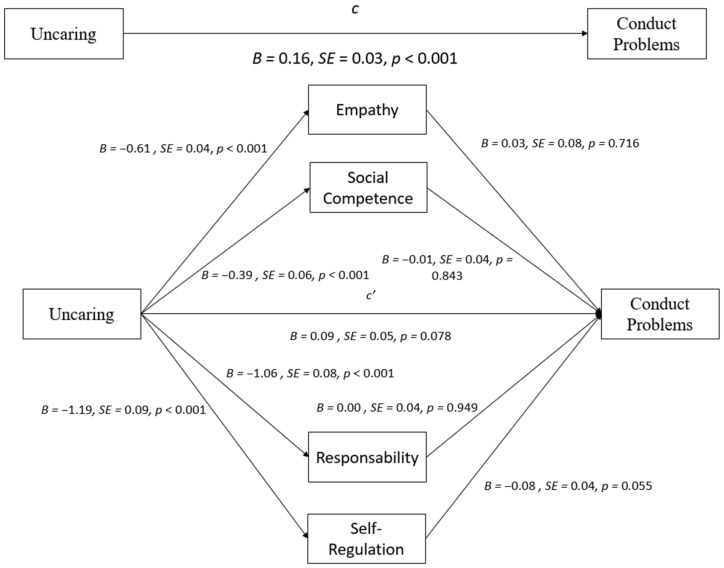
Mediation effects of strength variables on the relationship between the uncaring subscale and Conduct Problems.

With the uncaring subscale as the independent variable (Figure 3), bootstrapping analyses revealed the total effect on Conduct Problems, b = 0.16, *p* < 0.001. Empathy, responsibility, self-regulation, and social competence were not significant mediators in the relationship between the uncaring subscale and Conduct Problems (all *p* > 0.05). Individually, it was verified that the uncaring subscale predicted all strength and positive characteristics (all *p* < 0.001). However, none of these variables significantly predicted Conduct Problems (all *p* > 0.05). The results from multiple mediation analyses testing empathy, responsibility, self-regulation, and social competence as mediators between the uncaring subscale and Conduct Problems are presented in Table 3 and Figure 3.

## 4. Discussion

Over the last two decades, psychopathic traits in child and adolescent samples have been consistently associated with more severe and persistent patterns of Conduct Problems [11,70,71]. Given the increased risk that these traits seem to carry for the development of Conduct Problems throughout life and the importance of maximizing the potential of early prevention, it is of great interest to assess such traits as early as possible. For this reason, researchers have increasingly examined the role of callous–unemotional (CU) traits in children, gathering evidence that these traits may already be evident at preschool age [52,72,73]. According to Fanti et al. [74], it is necessary to understand the development of CU traits associated with different risk and protective factors for developing conduct problems in childhood and adolescence.

However, the factors associated with antisocial behavior have often been studied in isolation, even though they are not independent of each other and often interact with the same individual [75]. Thus, examining the sole influence of one factor will provide an insufficient explanation for Conduct Problems. Furthermore, although research in psychology has evolved over the last few decades, creating a movement towards greater emphasis on the assessment of individual strengths and positive characteristics (strength outcomes), the focus on risk factors or pathology diagnosis is still very evident [17,18,19,20]. Alongside the risk factors for pathology, assessing individual positive characteristics can provide a broader understanding of individuals’ abilities that can be used effectively to reduce the likelihood of developing disruptive behavior disorders.

Considering that previous research points to callous–unemotional (CU) traits as possible markers for a particularly severe presentation of Conduct Problems, eventually beginning in early childhood [76] and extending to childhood and adolescence, this study examines how strength characteristics could be related to CU traits, and possibly mediate the relationship between such traits and Conduct Problems in childhood.

Correlation analyses revealed the expected positive and significant association between the total CU traits score, as well as the callous and uncaring subscale scores and Conduct Problems. Additionally, correlation analyses revealed the expected significant but negative relationships between strength variables, namely social competence, self-regulation, empathy, and responsibility, with both CU traits and Conduct Problems. This preliminary analysis supported mediation analyses, which revealed that self-regulation significantly explains the relation between the callous dimension of the Inventory of Callous–Unemotional Traits and Conduct Problems; no other mediation effects were found. This indicates that self-regulation may act as a protective factor against the development of Conduct Problems. However, it is important to note that these findings are based on cross-sectional data and cannot establish causality. Longitudinal studies are necessary to investigate the developmental trajectories of CU traits and their influence on Conduct Problems.

The abovementioned finding suggests that characteristics of self-regulation as a strength variable, and more specifically, higher levels of self-regulation, may protect children with high CU traits from developing Conduct Problems. On the contrary, in line with Hadjicharalambous and Fanti’s [43] study, results support the idea that self-regulation deficits may contribute to the antisocial behavior of young people with high CU traits. Therefore, low self-regulation may interplay with low empathy and low remorse [4], leading someone to engage in antisocial behaviors. Prior works have already shown that impulsive individuals with deficits in self-control and attention are more likely to develop antisocial behavior due to their difficulties in regulating their behavior (e.g., [12]). Contrariwise, our results suggest that normative levels of self-regulation may be considered a protective factor, possibly enabling children to refrain from antisocial behavior despite their high levels of CU traits.

The results of this study should be considered alongside common methodological shortcomings in the field. First, cross-sectional research using mediation analyses cannot establish temporal precedence, so longitudinal data are needed to examine causation in these relationships. Additionally, it is necessary to consider the potential grouping of data since each teacher evaluated about 3 to 5 students. This clustering can introduce bias and lead to the non-independence of observations, which might affect the results. Future studies should account for this clustering effect, possibly using Multilevel Modeling techniques to control teacher-related variance. The way teachers approached classifying their students was not accounted for in our analyses, and systematic grouping was considered a potential source of measurement bias that could affect reliability [77]. Future studies could resort to a Multilevel Modeling approach to obtain unbiased parameter estimates and statistical inferences. To carry out a Multilevel Modeling approach, it is recommended to use cluster sizes of between 5 and 30 [78]. However, in this study, the number of cases evaluated per professor was insufficient to generate robust analyses, because, in our data, there were teachers with a minimum of one and a maximum of eight children evaluated. In addition, there was only one informant for the child’s Conduct Problems and individual characteristics (i.e., strength and positive characteristics), and it is known that a single informant may not provide a comprehensive view of Conduct Problems. This single-informant approach can lead to a one-sided perspective, potentially overlooking behaviors that occur outside the school environment. Therefore, this study must be replicated, but measures must be used from observational methods or data from multiple informants for the same variables, including, for example, assessments from the parents’ perspective. It is important to keep in mind that meta-analyses of multi-informant reports on child psychopathology (e.g., [79,80]) reveal a low correlation between parent and teacher reports (e.g., [81]). This may suggest that the information provided by parents and teachers covers different aspects of the child’s behavior, mainly because the observations are made in different environments (i.e., home vs. school). Thus, teachers’ reports are relevant and unique sources of information about children’s behavior. Teachers usually spend more time during the day with children than parents do and have the opportunity to observe them in various dynamics and interactions with other children and other adults [82]. In addition, teachers observe children in structured (e.g., classroom) and unstructured (e.g., playground) environments with their peers [83,84]. Finally, it should be noted that this study did not consider the assessment and/or analysis of confounding factors. These uncontrolled variables represent significant limitations as they may confound the observed relationships. Future studies should consider including such analyses to control potential confounders and provide a more comprehensive understanding of the variables influencing the outcomes. Examples of potential confounding factors include socioeconomic status, parental education levels, home environment, and prior exposure to interventions. Addressing these factors could enhance the robustness and validity of the findings.

Despite the above limitations, the results of this study add to those of others that contribute to a better understanding of the relationship between CU traits and Conduct Problems in preschool and school-age children. In fact, the trajectories of antisocial behavior begin to be investigated as early as the second year of life (e.g., [85]), and CU traits, measurable from the age of two, seem to play a central role in developing Conduct Problems. Identifying and assessing CU traits at an early age, as well as their associated factors, could have important implications for research into the structuring trajectories of psychopathic personalities, as well as for intervention with the potential to prevent the emergence of Conduct Problems. It should be noted that the same factors, depending on their expression (negative or positive), can act as risk or protective variables. At an individual level, social–emotional skills, specifically self-regulation, are important for the prosocial development of behavior, as they promote social skills and stable interpersonal relationships. As such, it is important to understand the potential effects of protective factors at different points in development. Thus, assessing and understanding positive characteristics can help inform strategies that address needs and challenges throughout development. As the percentage of children and young people who are considered at risk of developing conduct problems associated with emotional problems continues to rise, new ways of thinking about assessment and intervention services are needed. The public health approach and the well-known triangle of support model are recommended as a way of planning services for all children and adolescents in a given school or community population. Using this approach can help identify procedures and objectives for screening and assessment and help link the outcomes of screening and assessment to appropriate intervention services at the primary, secondary, and tertiary levels of prevention (e.g., see [86]). These strategies can involve training in social and emotional skills, including, for example, increasing emotional awareness, impulse control, and conflict management (e.g., [87,88]), encouraging parental involvement, and training in adopting methods to support the development of self-regulation.

## Figures and Tables

**Table 1:** Descriptive statistics for variables of interest.

Variables	Possible Range	N	Min	Max	Mean (SD)
Age	–	236	3	11	7.14 (1.99)
Education	–		–	–	–
Kindergarten	–	69	–	–	–
Elementary School	–	167	–	–	–
Total CU traits	(0–33)	215	0	27	8.76 (5.37)
Callousness	(0–18)	215	0	14	2.10 (2.89)
Uncaring	(0–15)	215	0	15	6.65 (3.31)
Conduct Problems	(0–10)	223	0	8	1.77 (1.62)
Self-Regulation	(0–39)	216	0	34	21.49 (5.60)
Empathy	(0–48)	216	2	18	11. 19 (2.71)
Social Competence	(0–39)	216	0	34	11.20 (3.05)
Responsibility	(0–30)	216	1	30	18.95 (5.13)

*Notes*. CU = callous–unemotional; SD = standard deviation.

**Table 2:** Correlations among variables of interest.

	1	2	3	4	5	6	7	8
1. Total of CU traits	–							
2. Callousness	0.85 **	–						
3. Uncaring	0.89 **	0.50 **	––					
4. Conduct Problems	0.32 **	0.21 **	0.334 **	–				
5. Self-Regulation	−0.74 **	−0.56 **	−0.71 **	−0.35 **	–			
6. Empathy	−0.72 **	−0.59 **	−0.74 **	−0.30 **	0.82 **	–		
7. Social Competence	−0.39 **	−0.24 **	−0.43 **	−0.21 **	0.55 **	0.52 **	–	
8. Responsibility	−0.74 **	−0.58 **	−0.70 **	−0.31 **	0.84 **	0.80 **	0.55 **	–

*Notes*. CU = callous–unemotional; ** *p* < 0.001.

**Table 3:** Indirect effects of CU traits on Conduct Problems.

Independent Variable	Mediated Effect	Point Estimate	SE	BCa 95% CI
Total CU traits				
	Empathy	−0.002	0.023	[−0.048, 0.044]
	Responsibility	−0.005	0.030	[−0.059, 0.056]
	Self-Regulation	0.057	0.034	[−0.009, 0.124]
	Social Competence	0.003	0.011	[−0.026, 0.016]
Callousness				
	Empathy	0.008	0.030	[−0.051, 0.067]
	Responsibility	0.005	0.044	[−0.076, 0.095]
	Self-Regulation	0.088 *	0.046	[0.005, 0.187]
	Social Competence	0.002	0.013	[−0.035, 0.015]
Uncaring				
	Empathy	−0.017	0.039	[−0.098, 0.056]
	Responsibility	−0.003	0.047	[−0.090, 0.093]
	Self-Regulation	0.089	0.051	[−0.011, 0.191]
	Social Competence	0.003	0.020	[−0.051, 0.028]

*Note.* SE = standardized error; BCa 95% CI = Bias-corrected and accelerated 95% confidence interval. * *p* < 0.05.

## Data Availability

The datasets presented in this article are not readily available because due to technical limitations. Requests to access the datasets should be directed to correspondence author.
